# Meiotic self-pairing of the *Psalidodon* (Characiformes, Characidae) iso-B chromosome: A successful perpetuation mechanism

**DOI:** 10.1590/1678-4685-GMB-2021-0084

**Published:** 2021-10-04

**Authors:** Duílio Mazzoni Zerbinato de Andrade Silva, Cristian Araya-Jaime, Masakane Yamashita, Mateus Rossetto Vidal, Claudio Oliveira, Fábio Porto-Foresti, Roberto Ferreira Artoni, Fausto Foresti

**Affiliations:** 1 Universidade Estadual Paulista (UNESP), Instituto de Biociências de Botucatu, Departamento de Biologia Estrutural e Funcional, Botucatu, SP, Brazil.; 2 Universidad de La Serena, Instituto de Investigación Multidisciplinar en Ciencia y Tecnología, La Serena, Chile.; 3 Universidad de La Serena, Departamento de Biología, Laboratorio de Genética y Citogenética Vegetal, La Serena, Chile.; 4 Hokkaido University, Faculty of Science, Department of Biological Sciences, Laboratory of Reproductive & Developmental Biology, Sapporo, Japan.; 5 Universidade Estadual Paulista (UNESP), Faculdade de Ciências, Departamento de Ciências Biológicas, Bauru, SP, Brazil.; 6 Universidade Federal de São Carlos (UFSCAR), Departamento de Genética e Evolução, São Carlos, SP, Brazil.; 7 Universidade Estadual de Ponta Grossa (UEPG), Departamento de Biologia Estrutural, Molecular e Genética, Ponta Grossa, PR, Brazil.

**Keywords:** Tetra fish, synaptonemal complex, SYCP3, meiotic silencing of unsynapsed chromatin (MSUC), fish meiosis

## Abstract

B chromosomes are non-essential additional genomic elements present in several animal and plant species. In fishes, species of the genus *Psalidodon* (Characiformes, Characidae) harbor great karyotype diversity, and multiple populations carry different types of non-essential B chromosomes. This study analyzed how the dispensable supernumerary B chromosome of *Psalidodon paranae* behaves during meiosis to overcome checkpoints and express its own meiosis-specific genes. We visualized the synaptonemal complexes of *P. paranae* individuals with zero, one, or two B chromosomes using immunodetection with anti-medaka SYCP3 antibody and fluorescence *in situ* hybridization with a (CA)_15_ microsatellite probe. Our results showed that B chromosomes self-pair in cells containing only one B chromosome. In cells with two identical B chromosomes, these elements remain as separate synaptonemal complexes or close self-paired elements in the nucleus territory. Overall, we reveal that B chromosomes can escape meiotic silencing of unsynapsed chromatin through a self-pairing process, allowing expression of their own genes to facilitate regular meiosis resulting in fertile individuals. This behavior, also seen in other congeneric species, might be related to their maintenance throughout the evolutionary history of *Psalidodon*.

## Introduction

Meiosis is a highly regulated process of reduced cell division occurring in germ cells, which allows for genetic recombination (Ma et al., 2014). During prophase I, homologous chromosomes are recognized and paired via synaptonemal complexes (SC), a protein structure formed between chromosomes. This structure is thought to mediate chromosome pairing, synapse, and even recombination. Meiotic silencing of unsynapsed chromatin (MSUC) significantly reduces transcriptional activity until the chromosomes reach their maximum state of pairing in the pachytene phase (Turner, 2015). MSUC seems to be involved in controlling nonhomologous recombination (McKee and Handel, 1993; Zhang et al., 2014) and activating the processes of cell death in cases of synapse failure (Faisal and Kauppi, 2016).

B chromosomes are non-essential additional genomic elements that (in most cases) arise from autosomal chromosomes and follow their own evolutionary path through various types of genome rearrangements, losing the ability to properly pair and recombine with A chromosomes (Houben et al., 2014). To perpetuate in the species, B chromosomes need to pair properly, avoiding meiosis disruption in a destructive manner. Cases where B chromosomes pair with each other to form bivalents, trivalents, and multivalents have been reported in literature (e.g. Pauls and Bertollo, 1983; Dias et al., 1998; Basheva et al., 2010; Serrano et al., 2016). This element frequently forms a univalent in cells with only one B chromosome, resulting in irregular meiosis. (Battaglia, 1964; Pauls and Bertollo, 1983; Shi et al., 1988; Dias *et al*., 1998; Basheva *et al*., 2010; Aquino et al., 2013; Pires et al., 2015; Serrano *et al*., 2016). Interestingly, iso-B chromosomes, reported in several species (Fletcher and Hewitt, 1988; Dias *et al*., 1998; Mestriner et al., 2000; Poletto et al., 2010; Valente et al., 2014; Pires *et al*., 2015), have the advantage of being composed of highly similar arms, allowing their perfect selfpairing, thus decreasing possible damage to cell division and increasing their chances of perpetuation (Battaglia, 1964; Mestriner *et al*., 2000; Serrano-Freitas et al., 2020).

The recently resurrected and expanded genus *Psalidodon* (Characiformes, Characidae) (Terán et al., 2020) includes species that formally belonged to the *Astyanax* genus, including *P. paranae* and *P. scabripinnis*, among others. These species harbor great karyotype diversity (Pazza et al., 2018) with several populations carrying different types of B chromosomes (Review in Silva et al., 2021). Among these species, the large metacentric B chromosome of *P. scabripinnis* was described as an isochromosome based on its self-pairing in meiotic cells and the symmetry of its arms as revealed by As51 satellite DNA mapping (Mestriner et al., 2000). This chromosome shares a common origin with the B chromosome of *P. paranae* (Silva *et al*., 2021), which is also an isochromosome based on the mapping of several repetitive DNAs (Silva *et al*., 2014, 2016, 2017). However, meiotic analyses have not yet been performed on this chromosome.

In this study, we analyzed the meiotic behavior of the B chromosome of *P. paranae* in individuals with one or two B chromosomes through synaptonemal complex immunodetection using an anti-medaka SYCP3 antibody and fluorescence *in situ* hybridization (FISH) using a (CA)_15_ microsatellite probe. We used an adaptation of the technique described by Araya-Jaime et al. (2015) to unveil the pairing mechanism employed by these elements to overcome the meiosis checkpoints and achieve evolutionary success in *Psalidodon*. We also examined the upregulation of a meiosis-specific gene present on the B chromosome (mutS homolog 4 - *msh4*) in the gonads of B-carrying individuals, to determine the pairing behavior of these extra elements.

## Material and Methods

### Sampling and measurement of B chromosome number

Ten females, seven males and one intersex individual of *P. paranae* from the population of Cascatinha stream, Botucatu, São Paulo, Brazil (22° 53’ 22.5” S, 48° 29’ 22.4” W) were analyzed. The intersex individual was identified by the presence of oocytes and spermatozoa under a microscopy analysis of the gonads. Previous studies reported the presence of B chromosomes in some individuals of this population (Maistro et al., 1994b; Porto-Foresti et al., 1997). The animals were collected in accordance with Brazilian environmental protection legislation (Collection Permission MMA/IBAMA/SISBIO-number 3245) and the procedures for fish sampling, maintenance, and analysis were performed in compliance with the Brazilian College of Animal Experimentation (COBEA) and approved (protocol 504) by the BIOSCIENCE INSTITUTE/UNESP ETHICS COMMITTEE ON THE USE OF ANIMALS (CEUA). The specimens were identified and deposited at the fish collection of the Laboratório de Biologia e Genética de Peixes, Botucatu, São Paulo, Brazil, under the voucher LBP19572 (*P. paranae*). The animals were anesthetized and dissected, and then mitotic chromosome preparations were obtained following the protocol described by Foresti *et al*. (1981). C-banding was performed according to the protocol described by Sumner (1972) to improve accuracy in measuring the number of B chromosomes in the samples. Chromosome preparations were stained for 5 minutes with 5% Giemsa solution in phosphate buffer (pH= 6.7).

### Synaptonemal complex analysis and fluorescence *in situ* hybridization

**Figure 1 -:**
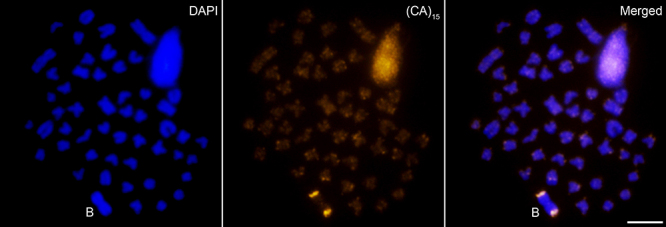
Metaphase of *P. paranae* (2n=50+B) after FISH with a (CA)_15_ probe. Chromosomes were counterstained with DAPI. Bar = 10 µm.

Synaptonemal complex preparations were performed according to Araya-Jaime et al. (2015) using immature males and females. The SCs were stained using silver nitrate and immunodetected using an anti-medaka SYCP3 primary antibody/FITC-labeled secondary antibody. Subsequent FISH technique on mitotic preparations and meiotic spreads (SC-FISH) was conducted according to the protocol described by Cioffi et al. (2011) using the (CA)_15_ oligonucleotide as probe. This probe was directly labeled with Cy3 during synthesis, as described by Kubat et al. (2008). This microsatellite was selected as a probe due to its high accumulation in the terminal region of both B chromosome arms at high visible sites (Silva et al., 2016) (Figure 1). Chromosomes were counterstained with 4’,6-Diamidino-2-phenylindole (DAPI) (Vector Laboratories, Burlingame, CA). Images were captured with a digital camera (Olympus DP90) attached to an Olympus BX61 epifluorescence photomicroscope and acquired using CellSens Dimension (Olympus). Image treatment, optimization of brightness and contrast were performed using the Adobe Photoshop CS4 program. Meiotic phases were identified according to Toscani et al. (2008), Ribagorda et al. (2019) and Spangenberg et al. (2019).

### Quantitative reverse transcription PCR (RT-qPCR)

Quantification of expression levels of *msh4* gene was performed using gonads from females and males with and without B chromosomes. We analyzed four ovaries 0B, six ovaries 1B, four testes 0B and three testes 1B. After dissection, tissues were immediately frozen in liquid nitrogen and stored at -70 °C. RNA was extracted using the TRIzol® Kit (Invitrogen), following the manufacturer’s instructions. Subsequently, the samples were treated with DNAseI (Thermo Fisher Scientific) and were checked on 1% agarose gel and on a 2100 Bioanalyzer® (Agilent) equipment. Only RNA samples with an A260/280 ratio of 1.8-2.0, A260/230 ratio > 2.0, and RIN > 7 were used for subsequent analysis. The cDNA for each sample was synthesized using the High-Capacity cDNA Reverse Transcription Kit® (Thermo Fisher Scientific, USA) using 1 μg per sample of total RNA, following the manufacturer’s instructions. The cDNA obtained was diluted in RNase-DNase free water for a 1:50 working solution. 

For RT-qPCR reactions, we used the primers for *msh4* gene designed by Silva et al. (2021). Quantitative PCR was performed on QuantStudio™ 12K Flex Real-Time PCR Systems (Thermo Fisher Scientific, USA). The reactions were performed in a final volume of 10 µL, with 1 μl of cDNA, 5 μl of Power SYBR™ Green PCR Master Mix (Thermo Fisher Scientific) and 1 μl of each 5 µM primer. Cycle conditions were 95 °C for 10 min; 45 cycles of 95 °C for 15 s, and 60 °C for 1 min. Target and reference genes were analyzed simultaneously in duplicates for two independent samples. The normalized relative expression quantity (NREQ) was determined by the 2^-∆∆Cq^ method (Livak and Schmittgen, 2001). The *msh4* expression levels were normalized using the hypoxanthine phosphoribosyltransferase 1 (*hprt1*) as the reference gene, with subsequent calibration to the average expression of the 0B group. The specificity of the PCR products was confirmed by dissociation curve analysis. 

Two-group comparisons were performed by the Gardner-Altman estimation plot method devised by Ho et al. (2019) following Gardner and Altman’s design (1986), as implemented on https://www.estimationstats.com/.

## Results

Meiocytes from five females (one 0B, three 1B and one not determined), five males (three 0B, one 1B and one 2B), and one intersex individual (0B) were analyzed. Overall, 48 meiocytes were analyzed, with an average of 4.8 cells analyzed per individual.

**Figure 2 -:**
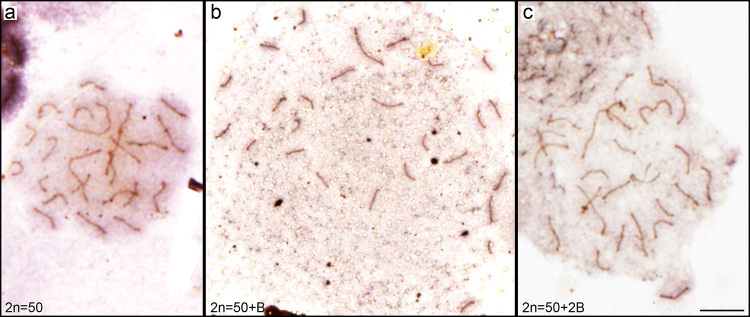
Detection of *P. paranae* synaptonemal complexes by silver nitrate staining (A, B, C). Diploid numbers of individuals are indicated in the lower left corner. Bar = 10 µm.

Silver nitrate staining of SCs revealed 25 bivalents in 0B individuals during the pachytene phase (Figure 2A), whereas in 1B individuals 26 completely paired SCs were observed (Figure 2B). The meiocytes of 2B individuals showed 26 SCs (Figure 2C). No differences between males and females were observed. 

**Figure 3 -:**
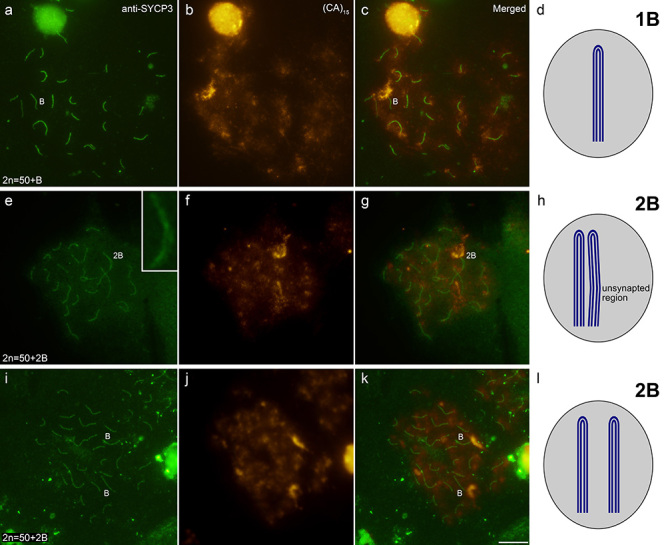
Detection of *P. paranae* synaptonemal complexes through immunodetection using an anti-medaka SYCP3 antibody (A, E, I) and FISH with a (CA)_15_ probe (B, F, J). Diploid number of individuals are indicated in the lower left corner (A, E, I). In the inset of e, note a region not fully associated in the lower half of the SC association formed by two self-paired B chromosomes. C, G, and K show the combination of images obtained by immunodetection and FISH. D, H, and L present an illustration of the meiotic behavior of the B chromosome. In all individuals, B chromosomes show a self-pairing behavior and in 2B individuals, they can stay close or separated. In the first case, they can appear not perfectly paired to each other. Bar = 10 µm.

Immunodetection analysis of SC revealed 25 bivalents in the 0B individuals (Figure S1). The SC-FISH assay revealed 25 fully synaptized bivalents in 1B individuals. As the B chromosome has conspicuous (CA)_15_ sites in both terminal regions (Figure 1), the assay also showed a SC marked by the (CA)_15_ probe in only one of the ends, corresponding to the self-paired B chromosome (Figure 3 A-C). In addition to 25 fully synaptized SCs, SC-FISH in 2B individual preparations showed a SC association marked on only one end by the (CA)_15_ probe, corresponding to two self-paired and close B chromosomes (Figure 3 E-G). A region not fully associated with the two SCs was observed in this structure (Figure 3E). In some pachytene preparations of 2B individuals, 25 normal bivalents plus two SCs marked by the (CA)_15_ probe were seen at only one of the ends, corresponding to two independently self-paired B chromosomes (Figure 3 I-K).

**Figure 4 -:**
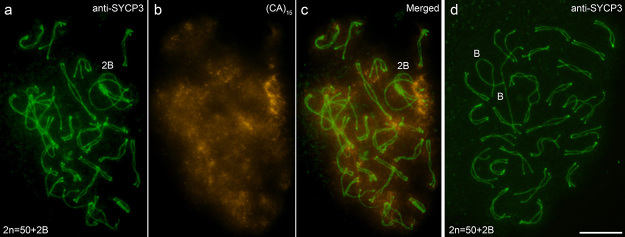
Diplotene images of *P. paranae* after immunodetection using anti-medaka SYCP3 antibody and SC-FISH assay using the (CA)_15_ probe (A, B, C) and the late-diplotene stages after immunodetection using the anti-medaka SYCP3 antibody (D). Diploid numbers of individuals are indicated in the lower left corners of images a and d. Note that in d, the B chromosomes are entirely dissociated, and are shown as univalents. Bar = 10 µm.

The meiotic behavior of two self-paired B chromosomes can be observed in Figure 4. In early diplotene, the formation of a “ring-like” figure can be observed with the chromosomes united at their ends (Figure 4 A-C). In more advanced stages of diplotene, complete dissociation of the B chromosomes shown as univalent was observed, apparently in a state of dissociation more advanced in relation to the chromosomes of the A complement (Figure 4D). The pairing patterns of *P. paranae* B chromosomes are illustrated in Figure 3 D, H, L. 

No variation was observed in the number of B chromosomes among the meiotic cells of each specimen. Thus, the 0B, 1B, and 2B individuals presented zero, one, and two B chromosomes in all their meiotic cells, respectively. No regions with failure in synapsis were observed among the chromosomes of the A complement in individuals of both sexes of *P. paranae* (Figures 2 and 3), suggesting the absence of proto-sex chromosomes at differentiation status (Murata et al., 2016). To the best of our knowledge, no sex-related chromosomal polymorphism has been described in the genus *Psalidodon*.

**Figure 5 -:**
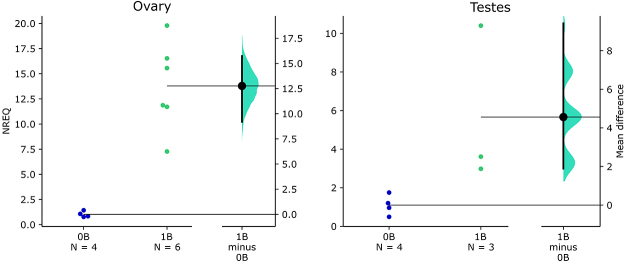
Gardner-Altman estimation plots showing *msh4* transcription levels in 0B and 1B individuals, as analyzed by RT-qPCR. Both groups are plotted on the left axes, and the mean difference (effect size) is plotted on a floating axe at the right as a bootstrap sampling distribution. The mean difference is depicted as a black dot, and the 95% confidence interval is indicated by the ends of the vertical error bar. NREQ = normalized relative expression quantity.

Transcription levels for the *msh4* gene were about the 12-fold effect size (mean differences) in 1B ovaries compared to those in 0B ones. In the testes, this difference was about 4-fold (Figure 5, Table S1). These results demonstrate upregulation of the *msh4* gene in the ovaries and testes of B-carrying individuals.

## Discussion

Meiotic self-pairing of iso-B chromosomes can allow them to advance through meiosis checkpoints and may be responsible for the success of their perpetuation (review in Clark et al., 2017; Silva et al., 2021). This behavior could allow B chromosomes without homologous pairs to circumvent the MSUC process. Thus, a selection pressure favoring the B iso-chromosomes would exist, as the MSUC process might lead to cell death in cases of synapse failure (Faisal and Kauppi, 2016). Our results thus demonstrate that B chromosomes of *P. paranae,* previously described as isochromosomes (Silva *et al*., 2014), can self-pair as observed in the B chromosome of its sister species, *P. scabripinnis* (Mestriner et al., 2000). After diverging for millions of years (Silva *et al*., 2021), both chromosomes still have self-pairing capacity, reinforcing the role of this behavior as one of the main mechanisms responsible for maintaining such chromosomes in the populations of *Psalidodon*. 

The success of B chromosomes is mainly related to their accumulation in populations due to transmission rates higher than 0.5, as expected by Mendelian laws. This process is called drive and occurs due to the patterns of B chromosome segregation (Camacho et al., 2005). As the techniques used here did not enable us to observe all the meiotic phases, we cannot verify whether the patterns of B chromosome segregation are influenced by other factors (Wu et al., 2019). The increased frequency of B chromosomes in the analyzed population (Goes, in prep.) might be related to their ability to circumvent the MSUC process and remain undetected or cause deleterious effects in carriers. 

B chromosomes are mainly composed of repetitive DNA sequences related to chromosome folding, recombination, meiotic pairing, and even disjunction in the germ line tissue (review in Garrido-Ramos, 2017); further, in several cases, pairing between B chromosomes was attributed to their homologous content (Pauls and Bertollo, 1983; Switonski et al., 1987; Kolomiets et al., 1988; Maistro et al., 1994a; De Brito Portela-Castro et al., 2001; Serrano et al., 2016). In this manner, repetitive DNA sequences symmetrically distributed on B chromosomes of *P. paranae* and *P. scabripinnis* (Mestriner et al., 2000; Silva et al., 2014, 2016, 2017; Artoni et al., 2015) would then be responsible for self-pairing and symmetry maintenance could be essential for perpetuating this advantageous behavior. 

In individuals with two B chromosomes, these elements are self-paired, even though the association between them is facultative (Figure 3 G, K). As discussed, self-pairing of B chromosomes may represent an adaptive advantage. Association of two self-paired B chromosomes might not be required to circumvent the MSUC process, because selfpaired B chromosomes do not have unsynapsed regions. The association between two B chromosomes observed here appears to result from their homologous content recognition and/or their physical proximity to specific territories inside the nucleus (Schemczssen-Graeff et al., 2020). When B chromosomes are associated, we observe synaptic regions forming between chromatids (Figure 4A), as the employed technique preserves chromosomal associations and avoids the “squash” procedure (Araya-Jaime et al., 2015).

Self-pairing of B chromosomes can favor homogenization of their sequences through regular recombination (Rice, 1994; Basheva et al., 2010). The low diversification of ITS1 and ITS2 regions on the B chromosome of *P. paranae* was attributed to this phenomenon by Silva et al. (2014). Regular recombination can also be responsible for the conservation of protein-coding sequences observed on the B chromosomes of *P. paranae* and *P. scabripinnis* (Silva *et al*., 2021). This conservation was indicated by the presence of a few B-specific SNPs and complete coding regions on these chromosomes (Silva *et al*., 2021). Similarly, natural selection has preserved the sequences of apparently intact and functional c-KIT on the B chromosome of foxes (Graphodatsky et al., 2005; Yudkin et al., 2007). In this case, recombination of B chromosomes was considered a protective mechanism for copies of this gene against mutational meltdown and degeneration (Basheva *et al*., 2010).

Unpaired B chromosomes can be silenced at meiosis (Serrano et al., 2016), as observed in unpaired regions in the fungus *Neurospora crassa* (Sordariales, Sordariaceae) (Shiu and Metzenberg, 2002) and in mice (Turner et al., 2005). Self-pairing of B chromosomes, observed in *P. paranae* (this study) and in *P. scabripinnis* by Mestriner et al. (2000), could allow them to escape from silencing epigenetic modifications of the MSUC process, resulting in the transcriptional activity of these chromosomes (Serrano *et al*., 2016). Accordingly, Silva et al. (2021) identified the expression of B-specific transcripts of important genes related to the cell cycle and gonadal development in the ovaries of both species, revealing that the B chromosomes are not silenced during meiosis.

Among them, the *msh4* gene, a meiosis-specific homologue of the bacterial MutS protein, is essential for meiotic recombination and proper segregation of homologous chromosomes. In mice, meiosis is severely disrupted in MSH4 mutant males and females, resulting in infertility (Kneitz et al., 2000; Dai et al., 2017). We consider that transcription of the *msh4* gene on the B chromosomes of *P. paranae* and *P. scabripinnis* helps ensure the required conditions for the proper division of cells carrying extra elements with atypical pairing. We observed upregulation of this gene in the ovaries and testes of 1B individuals of *P. paranae*, highlighting their role during the divisions leading to gonad formation in both sexes.

In conclusion, this study shows that the B chromosomes of *P. paranae* and *P. scabripinnis* can express their own meiosis-specific genes by escaping the MSUC through meiotic self-pairing. This ancient behavior results in regular meiosis and fertile individuals, allowing the evolutionary success and maintenance of B chromosomes throughout the evolutionary history of *Psalidodon*. Extension of these analyses beyond the iso-B chromosomes of *Psalidodon* is necessary to comprehend how non-isochromosome behavior perpetuates in this genus. The use of controlled crosses could provide information about the segregation patterns and transmission rates of the B chromosome in *P. paranae*.

## Supplementary material

The following online material is available for this article:

Figure S1 - Detection of *P. paranae* synaptonemal complexes through immunodetection using anti-medaka SYCP3 antibody.

Table S1 - Relative transcription levels for the gene *msh4* in B-carrying and B-lacking individuals.
