# Supplementary material for: Meiotic self-pairing of the Psalidodon (Characiformes, Characidae) iso-B chromosome: A successful perpetuation mechanism
Source: Genet Mol Biol. 2021 Oct 4;44(3):e20210084. doi: 10.1590/1678-4685-GMB-2021-0084 (PMC8495774; doi:10.1590/1678-4685-GMB-2021-0084)
Supplement: Figure S1 - [file 1415-4757-GMB-44-3-e20210084-s1.pdf]

Genetics and Molecular Biology
https://doi.org/10.1590/1678-4685-GMB-2021-0084

Supplementary Material to "Meiotic self-pairing of the *Psalidodon* (Characiformes, Characidae) iso-B chromosome: A successful perpetuation mechanism"

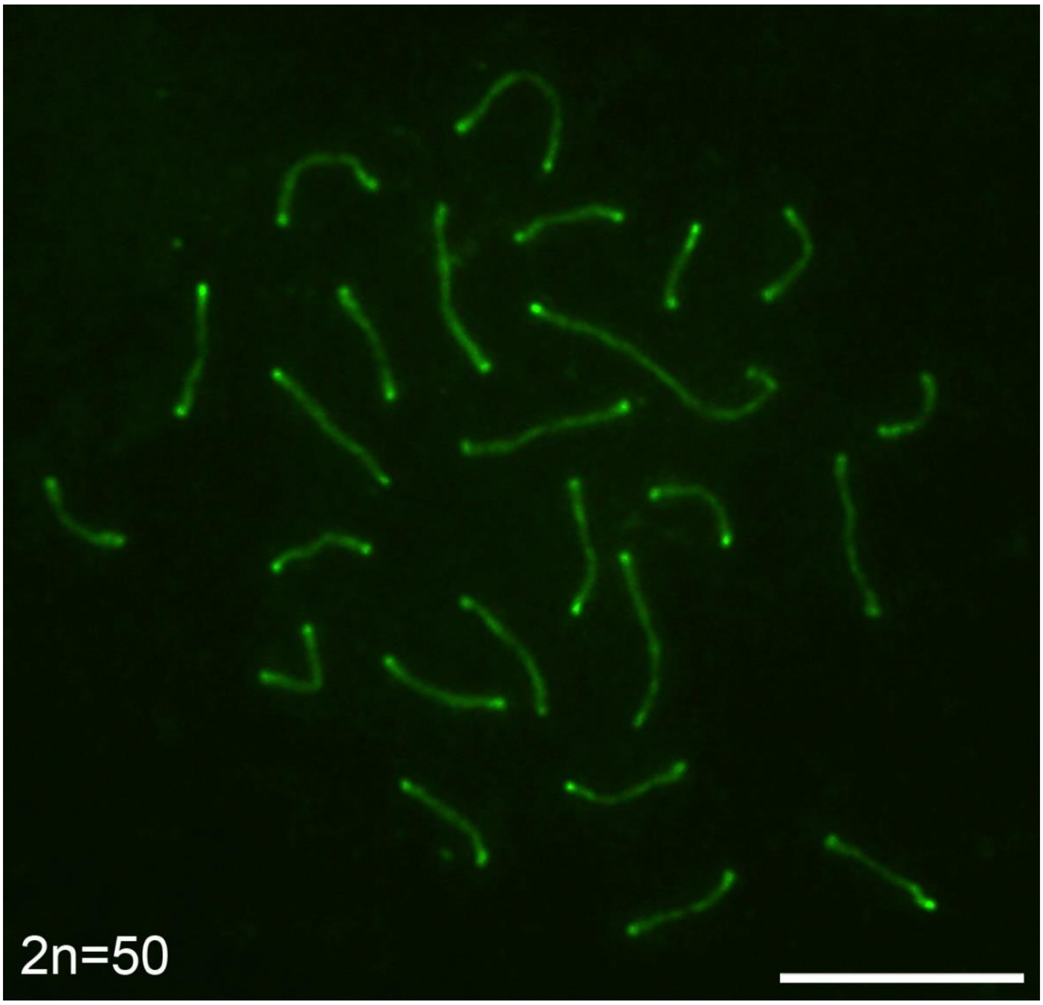

Figure S1 – Detection of *P. paranae* synaptonemal complexes through immunodetection using anti-medaka SYCP3 antibody. The diploid number of the individual is indicated in the lower left corner. Bar = 10 µm.
