# Supplementary material for: Meiotic self-pairing of the Psalidodon (Characiformes, Characidae) iso-B chromosome: A successful perpetuation mechanism
Source: Genet Mol Biol. 2021 Oct 4;44(3):e20210084. doi: 10.1590/1678-4685-GMB-2021-0084 (PMC8495774; doi:10.1590/1678-4685-GMB-2021-0084)
Supplement: Table S1 - [file 1415-4757-GMB-44-3-e20210084-s2.pdf]

Genetics and Molecular Biology
https://doi.org/10.1590/1678-4685-GMB-2021-0084

Supplementary Material to "Meiotic self-pairing of the *Psalidodon* (Characiformes, Characidae) iso-B chromosome: A successful perpetuation mechanism"

Table S1 – Relative transcription levels for the gene *msh4* in B-carrying and B-lacking individuals. Bs = Number of B chromosomes in the sample. Cq = Cycle of quantification. NREQ = Normalized relative expression quantity. SD = Standard deviation.

Data form Ovary

Bs	Sample	Target	Cq	Cq Mean	Delta Cq	NREQ	Group	NREQ Mean	SD
0	1	HPRT	30.03	29.84			0B	1.03	0.30
0	1	HPRT	29.64	29.84			1B	13.79	4.41
0	1	MSH4	33.35	33.57	3.73	1.07			
0	1	MSH4	33.79	33.57					
0	5	HPRT	23.62	23.68					
0	5	HPRT	23.74	23.68					
0	5	MSH4	26.87	27.00	3.32	1.43			
0	5	MSH4	27.12	27.00					
0	9	HPRT	30.08	30.03					
0	9	HPRT	29.99	30.03					
0	9	MSH4	34.44	34.12	4.08	0.84			
0	9	MSH4	33.80	34.12					
0	69	HPRT	21.41	21.56					
0	69	HPRT	21.70	21.56					
0	69	MSH4	25.65	25.76	4.20	0.77			
0	69	MSH4	25.88	25.76					
1	4	HPRT	25.48	25.44					
1	4	HPRT	25.40	25.44					
1	4	MSH4	24.86	24.97	-0.47	19.79			
1	4	MSH4	25.08	24.97					
1	6	HPRT	23.06	23.15					
1	6	HPRT	23.23	23.15					
1	6	MSH4	23.39	23.43	0.28	11.71			
1	6	MSH4	23.47	23.43					
1	8	HPRT	25.09	25.05					
1	8	HPRT	25.01	25.05					
1	8	MSH4	24.92	24.84	-0.21	16.53			
1	8	MSH4	24.75	24.84					
1	70	HPRT	29.17	29.28					
1	70	HPRT	29.39	29.28					

Bs	Sample	Target	Cq	Cq Mean	Delta Cq	NREQ	Group	NREQ Mean	SD
1	70	MSH4	30.37	30.25	0.97	7.27			
1	70	MSH4	30.13	30.25					
1	71	HPRT	28.64	28.60					
1	71	HPRT	28.55	28.60					
1	71	MSH4	28.81	28.86	0.27	11.87			
1	71	MSH4	28.92	28.86					
1	72	HPRT	28.96	28.91					
1	72	HPRT	28.87	28.91					
1	72	MSH4	28.80	28.79	-0.13	15.56			
1	72	MSH4	28.77	28.79					

Data from testes

Bs	Sample	Target	Cq	Cq Mean	Delta Cq	NREQ	Group	NREQ Mean	SD
0	61	HPRT	27.40	27.53		1.75	0B	1.10	0.52
0	61	HPRT	27.66	27.53		0.96	1B	5.66	4.11
0	61	MSH4	30.55	30.59	3.06	1.20			
0	61	MSH4	30.64	30.59		0.49			
0	62	HPRT	26.25	26.20		3.61			
0	62	HPRT	26.14	26.20		10.40			
0	62	MSH4	29.78	30.12	3.93	2.98			
0	62	MSH4	30.46	30.12					
0	66	HPRT	27.60	27.64					
0	66	HPRT	27.69	27.64					
0	66	MSH4	31.33	31.25	3.61				
0	66	MSH4	31.17	31.25					
0	68	HPRT	25.66	25.61					
0	68	HPRT	25.55	25.61					
0	68	MSH4	30.38	30.49	4.89				
0	68	MSH4	30.61	30.49					
1	63	HPRT	29.58	29.51					
1	63	HPRT	29.43	29.51					
1	63	MSH4	31.51	31.53	2.02				
1	63	MSH4	31.54	31.53					
1	64	HPRT	27.88	27.90					
1	64	HPRT	27.92	27.90					
1	64	MSH4	28.45	28.39	0.49				
1	64	MSH4	28.33	28.39					
1	73	HPRT	26.34	26.26					
1	73	HPRT	26.19	26.26					
1	73	MSH4	28.22	28.56	2.30				
1	73	MSH4	28.90	28.56					
